# Transcatheter Edge-to-Edge Mitral Valve Repair Device Embolization into the Right Lower Pulmonary Vein

**DOI:** 10.1016/j.jaccas.2026.107270

**Published:** 2026-03-06

**Authors:** Dennis D. Kumi, Pablo Barzallo, Kumaran S. Senthil, Kyle Coombes, Michael Z. Grzeskowiak, Sudhir Mungee, Ashvarya Mangla

**Affiliations:** Department of Medicine, University of Illinois College of Medicine at Peoria, Peoria, Illinois, USA

**Keywords:** embolization, MitraClip, periprocedural complication, percutaneous retrieval, pulmonary vein

## Abstract

**Background:**

Excellent efficacy and safety have led to an increase in transcatheter edge-to-edge repair (TEER) procedures. MitraClip embolization into the pulmonary veins has not yet been reported in the literature.

**Case Summary:**

A 77-year-old man in cardiogenic shock with severe mitral regurgitation underwent a MitraClip procedure. Deployment of a second MitraClip was complicated by knotting of the locking threads. Attempts to release these threads led to embolization of the MitraClip into the right lower pulmonary vein. Transesophageal echocardiography–guided retrieval using a gooseneck snare through a multipurpose catheter led to safe clip removal.

**Discussion:**

MitraClip embolization is a rare event that complicates up to 0.1% of cases. This is to our knowledge the first case report on embolization into the pulmonary veins. Lock-thread failure as the mechanism for embolization is uncommon. Our retrieval assembly, consisting of a gooseneck snare through a long multipurpose catheter then through the MitraClip steerable catheter, provided adequate reach, torque, and maneuverability for safe retrieval.

**Take-Home Messages:**

The pulmonary veins are a rare location for MitraClip embolization. Appropriate catheter selection and optimal grasp orientation are key to event-free retrieval.

## Visual Summary

**Figure:**
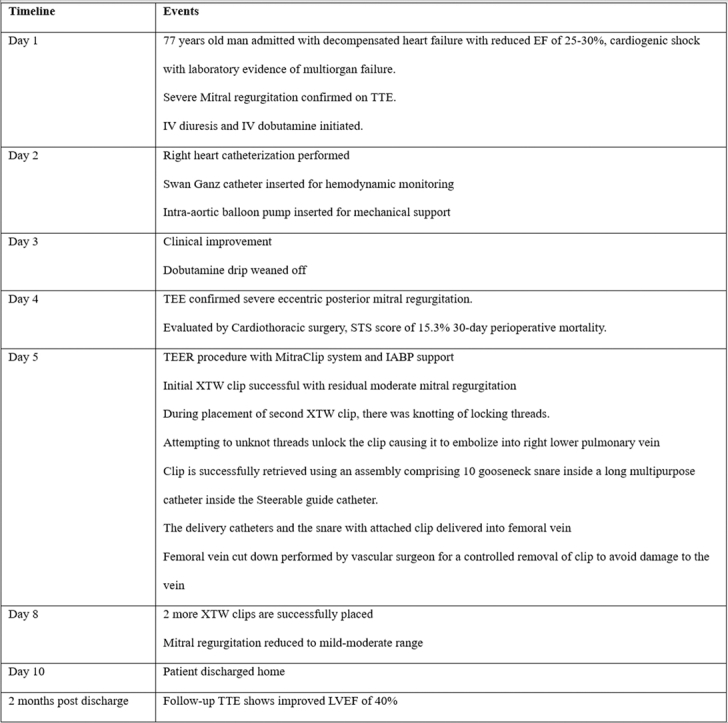

## History of Presentation

A 77-year-old man was admitted after 3 days of progressive dyspnea, orthopnea, dizziness, and lethargy. On examination, he was hypotensive (blood pressure: 91/52 mm Hg), tachycardic (heart rate: 115 beats/min), and hypoxemic (SPO_2_ of 89%), requiring supplemental oxygen in the emergency room. He had bilateral pedal edema, bilateral lung crackles, elevated jugular venous pressure, and cold and clammy extremities.

**Take-Home Messages**

• Pulmonary veins are a rare but important location for TEER device embolization.
• A key to safe and timely transcatheter retrieval of embolized TEER devices lies in appropriate catheter selection and in obtaining an optimal grasp orientation.

## Past Medical History

The patient's medical history included ischemic cardiomyopathy after having received an implantable cardioverter-defibrillator, paroxysmal atrial fibrillation, chronic obstructive pulmonary disease, and chronic moderate secondary mitral regurgitation.

## Investigations

Initial laboratory work-up showed elevated creatinine, liver enzymes, lactate, and N-terminal pro–B-type natriuretic peptide levels. Other tests, including thyroid-stimulating hormone, complete blood count, and infectious/sepsis work-up, were unremarkable. Chest x-ray showed bilateral cephalization with increased interstitial opacities bilaterally. Transthoracic echocardiography showed severely reduced systolic function; left ventricular ejection fraction was 25% to 30%, with severe mitral regurgitation, elevated right ventricular systolic pressure of 56 mm Hg, and dilated inferior vena cava. Right heart catheterization showed postcapillary pulmonary hypertension, with right atrial pressure of 12 mm Hg, mean pulmonary artery pressure of 41 mm Hg, pulmonary capillary wedge pressure of 45 mm Hg with V-wave of 57 mm Hg, and cardiac index of 2.1 L/m^2^.

## Diagnosis

The patient was diagnosed with cardiogenic shock, severe mitral regurgitation, and multiorgan dysfunction syndrome.

## Management

**Figure 1:**
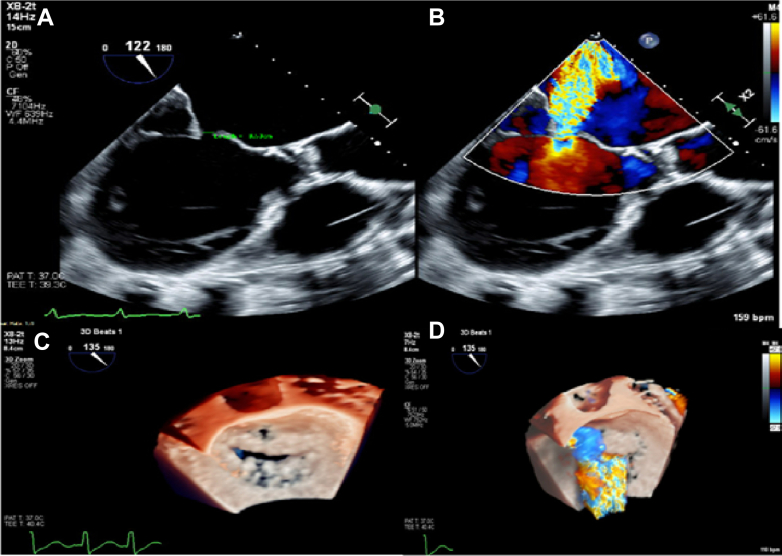
Transesophageal Echocardiography Images Confirming Severe Eccentric Mitral Regurgitation (A) Overriding anterior leaflets due to posterior leaflet tethering. (B) Color Doppler flow with severe regurgitation. (C) Three-dimensional image of coaptation gap during mitral valve closure in systole. (D) Three-dimensional color Doppler of mitral regurgitation in systole.

Initial medical treatment included hemodynamic support with intravenous dobutamine drip and diuresis with intravenous Lasix (furosemide) drip. On day 2 of admission, given persistent shock, the patient underwent placement of intra-aortic ballon pump for mechanical support. Dobutamine was weaned off on day 3. Improved volume status was achieved after 12 L net negative fluid balance on day 4 of aggressive inotrope-supported diuresis. Transesophageal echocardiography on day 4 of admission confirmed severe posteriorly directed mitral regurgitation with functional anterior leaflet flail due to restricted posterior leaflet. Details of the severity of mitral regurgitation are depicted in Figure 1 and Video 1. A shared decision was made to proceed with TEER. The patient's Society of Thoracic Surgeons score predicted a postoperative 30-day mortality of 15.3%; hence, he was deemed an unfavorable candidate for valve surgery.

## MitraClip Procedure

**Figure 2:**
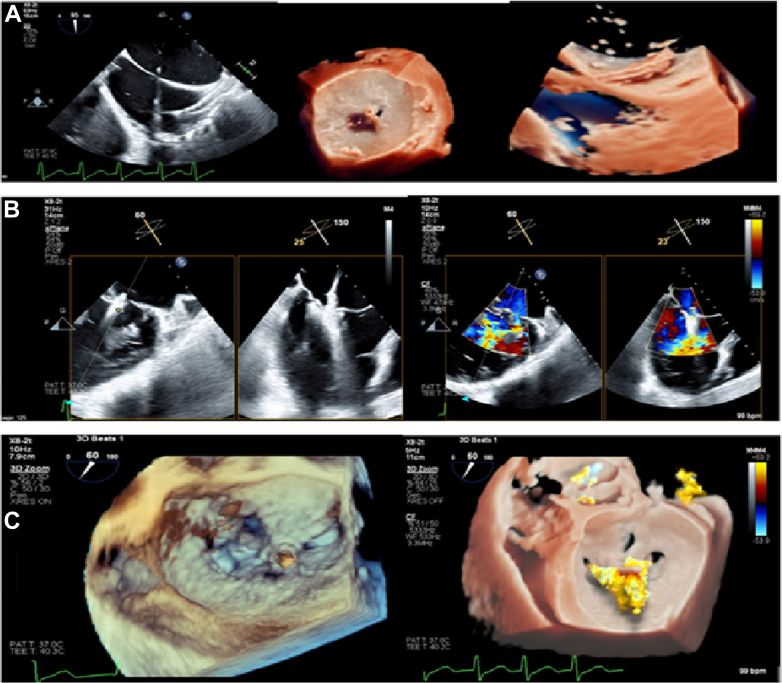
Echocardiographic Images Showing Different Stages of the Initial XTW MitraClip Placement (A) Two- and three-dimensional images showing transseptal puncture during TEER procedure: (B) Two-dimensional and color Doppler images showing successful deployment of the first XTW MitraClip with moderate residual mitral regurgitation. (C) Three-dimensional images showing successful deployment of the first XTW MitraClip with moderate residual mitral regurgitation. TEER = transcatheter edge-to-edge repair.

The MitraClip procedure was performed with the patient on intra-aortic ballon pump support. Right femoral vein access was obtained. Upon successful transseptal puncture, a good P2-A2 scallop grasp with adequate tissue bridge was obtained, and the first XTW MitraClip was successfully deployed using the G4 MitraClip delivery system. There was immediate improvement in systolic blood pressure of 10 mm Hg. There was residual moderate to severe regurgitation. Procedure details are depicted in Figure 2 and Video 2.

## Placement of Second MitraClip and Clip Embolization

**Figure 3:**
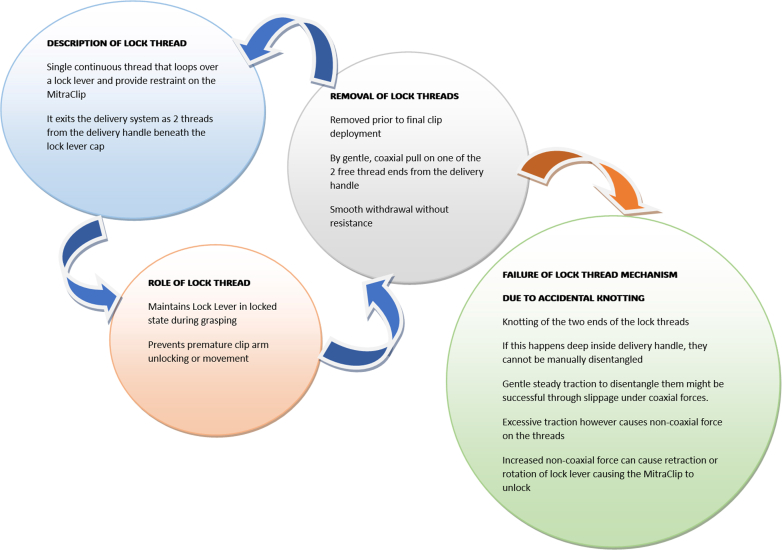
Simplified Schematic Diagram Depicting the Function, Normal Removal, and Mechanism of Functional Failure of the Lock Thread in the G4 MitraClip Delivery System

**Figure 4:**
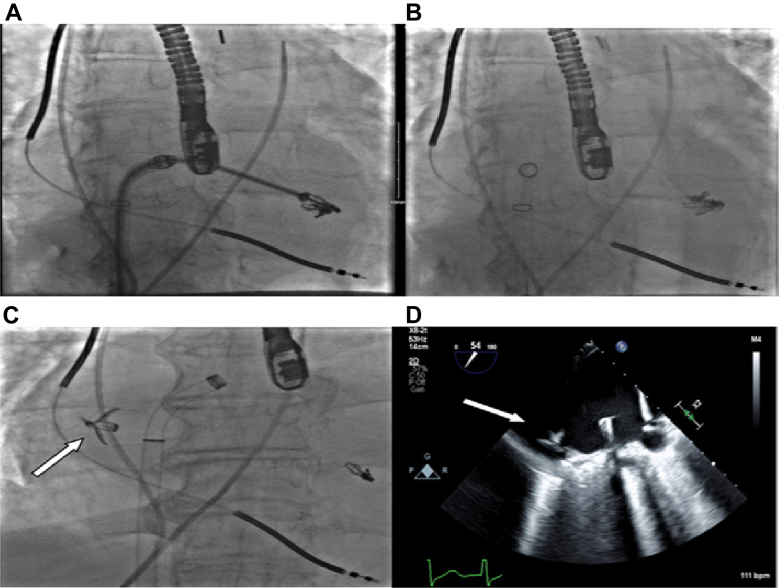
Fluoroscopic and Echocardiographic Images Depicting Events Leading to Embolization of MitraClip Into Right Lower Pulmonary Vein Fluoroscopic images showing (A) second MitraClip deployment next to the first MitraClip, with the delivery system across the mitral valve; (B) the delivery system retracted, with the second clip unstable next to the first clip on fluoroscopy; and (C) the second MitraClip embolized into the right lower pulmonary vein (arrow). (D) Two-dimensional echocardiogram showing the embolized MitraClip in the lower right pulmonary vein.

We proceeded to deploy a second XTW MitraClip. After confirming good grasp lateral to the first clip, the second XTW MitraClip was locked securely in place, but the lock threads became knotted deep inside the delivery system while they were being pulled out and could not be removed. The schematic diagram in Figure 3 gives a graphical depiction of the lock-thread function and potential mechanism of lock-thread failure. The decision was taken to proceed with release of the MitraClip from the delivery catheter and apply gentle traction to unknot the threads. An attempt to gently tug on the threads to unknot them unlocked the MitraClip, and the clip became loosely attached via the grippers. Under the current of the regurgitant stream, the clip embolized into the steering guide catheter, but for only a few seconds before it further embolized into the right lower pulmonary vein after coming loose from the threads (Figure 4, Video 3).

## Retrieval of Embolized MitraClip

**Figure 5:**
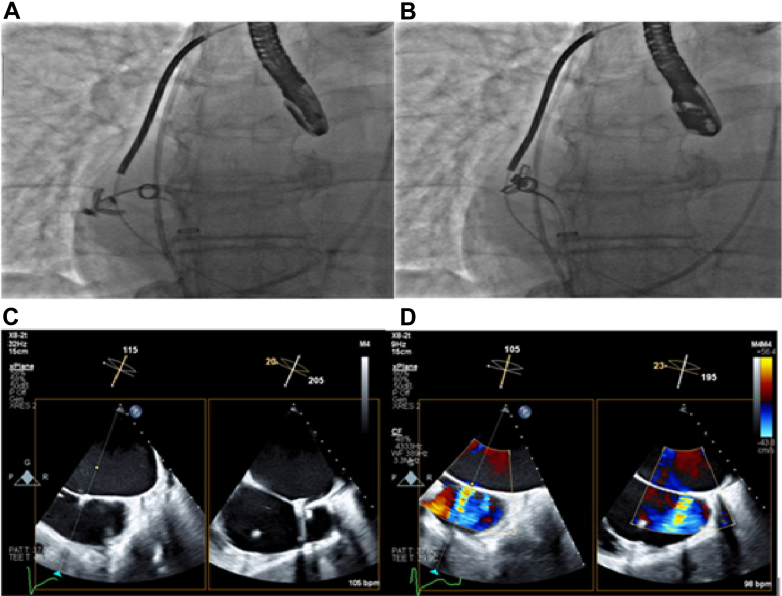
Fluoroscopic and Echocardiographic Images Depicting Events During Retrieval of Embolized MitraClip From Right Lower Pulmonary Vein (A) Fluoroscopic image showing a 10 mm gooseneck snare through multipurpose catheter directed at the embolized MitraClip. (B) Fluoroscopic image showing the gooseneck snare securely holding the neck of the MitraClip and pulled inside the steerable catheter. (C) Two-dimensional echocardiogram showing the MitraClip at the tip of the catheter and the snare assembly being pulled across the interatrial septostomy. (D) Two-dimensional TEE with color Doppler showing iatrogenic atrial septal defect after the clip and catheters have been pulled out of the interatrial septostomy. TEE = transesophageal echocardiography.

A 10 mm gooseneck snare was advanced directly through the steerable guide catheter, but the snare fell short and could only grasp one of the gripper arms. This was deemed unsafe, and the approach was abandoned. A long, multipurpose diagnostic catheter was introduced through the steerable guide catheter, and then the 10-gooseneck snare advanced through it. The embolized clip was successfully snared at the neck and pulled back to the mouth of the steerable guide, then the entire delivery system with the snared clip was pulled across the atrial septostomy into the right atrium. The entire assembly was safely retrieved into the femoral vein. There was no evidence of leaflet damage or perforation at the end of the procedure. With exception of an iatrogenic atrial septal defect due to our transseptal puncture, the interatrial septum was intact. Vascular surgery removed the clip with a femoral venous cut down to avoid damage to the femoral vein. Procedure details are depicted in Figure 5 and Video 4. Details on interventional equipment are provided in Table 1.

**Table 1:** MitraClip Procedure Equipment List

Imaging	• Transesophageal echocardiogram (GE Healthcare) o 3D TEE probe
Vascular access	• Ultrasound machine (GE Healthcare) • 5-F platinum-tipped micropuncture needle and wire • 0.035-inch × 150-cm, 3-mm Medline guidewire, J wire • 5-F 10-cm Pinnacle sheath (Terumo) in left femoral artery • 8-F 10-cm Pinnacle sheath (Terumo) in right femoral vein
Transseptal puncture	• Swartz braided transseptal guiding introducer 8.6-F SL-1 sheath (St Jude Medical) • NRG transseptal needle, large curve C1, 71-cm (Baylis Medical) • 0.035-inch × 210-cm Go2 wire (Merit Medical) • 0.035-inch × 260-cm, 3-mm J-tip Amplatz extra stiff guidewire (Cook) • 20-F Coons taper dilater (Cook)
MitraClip device implantation	• MitraClip G4 system (steerable guide catheter and clip delivery system) (Abbott) • MitraClip G4 XTW clip (Abbott)
MitraClip retrieval	• Amplatz Goose Neck snare kit (Medtronic) • 11-F 10-cm Pinnacle sheath (Terumo) • Multipurpose diagnostic catheter
Vascular access closure	• Perclose ProStyle (Abbott) • 5-F Mynx grip closure device (AccessClosure)

TEE = transesophageal echocardiography.

## Outcome and Follow-Up

Three days later, 2 more XTW MitraClips were successfully deployed with improvement of mitral regurgitation to the mild to moderate range. Transesophageal echocardiography performed 2 months later showed improved left ventricular ejection fraction to 40% with mild-moderate mitral regurgitation. Clinical response was excellent, and during the 6-month follow-up visit, the patient continued to feel much better, with no rehospitalizations.

## Discussion

Transcatheter edge-to-edge repair (TEER) with the MitraClip system has become a preferred intervention for high-risk patients with mitral regurgitation based on established efficacy, favorable safety profile, and excellent procedural success rates. Overall perioperative complications are low and include single-leaflet device attachment (1.7% to 4.8% of cases), pericardial effusion/tamponade in (<1%), and, rarely, device embolization (0.1% to 0.3%).^1^^,^^2^ Clip embolization is a rare event that complicates TEER, and documented anatomic destinations of embolized clips include the left atrium, left ventricle, aortic root, sinus of Valsalva, descending thoracic aorta, and coronary artery ostium.^3^^,^^4^ These events can lead to hemodynamic instability, valvular dysfunction, or coronary ischemia, often necessitating urgent intervention.^5^ Retrieval strategies have included percutaneous snaring, surgical extraction, or hybrid approaches and are determined by the device's location and the patient's clinical status.^6^

Common mechanisms of MitraClip embolization include incomplete leaflet grasping, progression from single-leaflet device attachment, excessive tension or manipulation during deployment, and deployment onto structurally inadequate leaflets such as those that are flail, short, or heavily calcified.^3^^,^^6^ To our knowledge, this is the first documented case of a MitraClip embolizing into a pulmonary vein, thereby expanding the known anatomical destinations for device migration during TEER. Most clip embolization seems to have a forward flow trajectory toward the left ventricle and aortic sinus. Rarely, torrential mitral regurgitation with high shear flow may contribute to atrial-level clip embolization.^6^^,^^7^ In our patient, an initial atrial-level embolization was due to excessive tension from the operator in an attempt to unknot the lock threads, which inadvertently caused the MitraClip to unlock. Additionally, the combination of severe mitral regurgitation and significantly elevated left atrial pressures likely generated hemodynamic forces that facilitated a posterior embolization into the pulmonary venous system after the threads came loose instead of the usual forward direction toward the left ventricle, as described in most case reports.

Lock-thread failure as the mechanism of embolization, as in our case, is not commonly encountered. Currently, most institutions use the G4 MitraClip system. This system has lock threads (lock lines) that run over a metallic hair-loop harness connected to the MitraClip. At the end of leaflet grasping and locking of the clip, the lock threads are removed before the MitraClip is detached from the delivery system. The step of removing the lock threads is often very routine, but these threads can in rare cases can get entangled. In most cases, knotting of the threads occurs outside the delivery system, where they are accessible to manual correction. In rare instances such as ours, knotting happens deep inside the delivery system. In these cases, gentle tugging can unknot them.^8^ The danger with this maneuver however, is that the hair-pin loop harness through which these threads run can be pulled, and if this pulling is strong enough, the MitraClip will be unlocked. The current-generation G5 MitraClip system overcomes this complication by eliminating the lock threads entirely from its design.

Percutaneous retrieval has become widely used in the retrieval of embolized clips. There is currently no standardized approach, and various operators have used different strategies.^9^^,^^10^ In our case, there were 3 imminent technical challenges. 1) timeliness of retrieval: any delays could result in intrusion of the clip deeper into the pulmonary vein, or conversely, its extrusion toward the left ventricle and aortic sinus, posing even greater risk. 2) Safe grasp orientation to avoid the free-floating grippers from catching onto delicate intracardiac structures. In this regard, grasping the neck of the clip was deemed the most optimal, as this position gives the most stable grasp and allows the operator to safely orient the grippers to minimize injury. 3) Selecting the appropriate catheter. The assembly we used (a snare in a long multipurpose catheter in the MitraClip steerable guide) offered a quick, safe, and reliable approach that provided excellent reach, adequate torque, and optimal maneuverability.

**Visual Summary:**
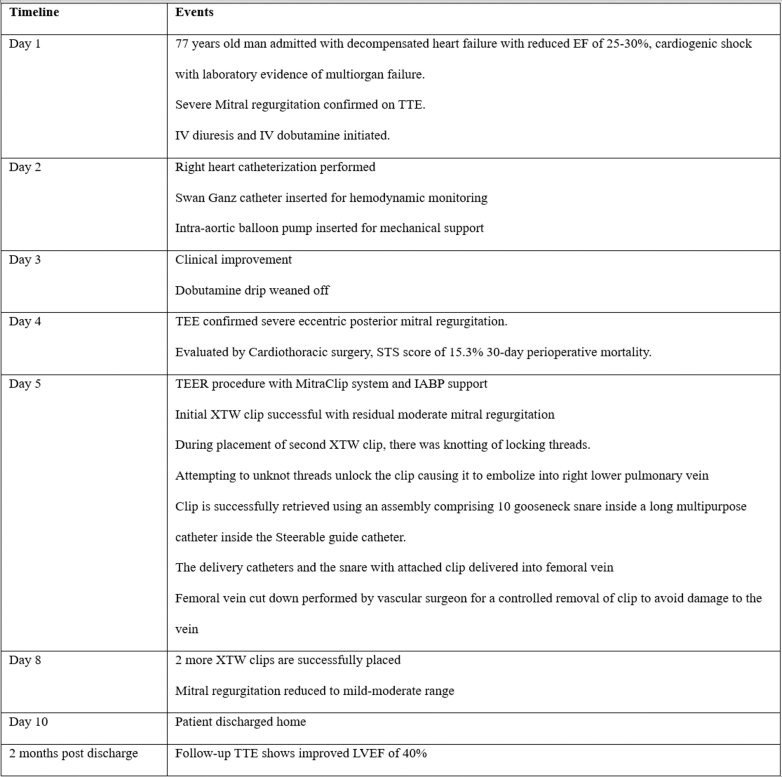
Timeline of the Case EF = ejection fraction; IABP = intra-aortic balloon pump; IV = intravenous; LVEF = left ventricular ejection fraction; STS = Society of Thoracic Surgeons; TEE = transesophageal echocardiography; TEER = transcatheter edge-to-edge repair; TTE = transthoracic echocardiography.

## Conclusions

This case broadens the clinical understanding of MitraClip-related embolization, highlights a unique anatomical trajectory, and emphasizes the importance of imaging-guided retrieval techniques in such cases. Our report adds to the growing body of literature on TEER complications and informs future management strategies. Important factors for a reliable, safe, and quick retrieval include appropriate catheter selection, optimal grasp point and orientation, and timeliness of retrieval guided by real-time imaging. A retrieval assembly consisting of a gooseneck snare through a long multipurpose catheter and through the MitraClip steerable catheter provides adequate reach, torque, and maneuverability.

## Funding Support and Author Disclosures

The authors have reported that they have no relationships relevant to the contents of this paper to disclose.

## Appendix

Video 1 Transesophageal Echocardiographic 2D and Color Doppler Videos Demonstrating Severe Eccentric Mitral Regurgitation

Video 2 Transesophageal Echocardiographic 3D Color Doppler Video Showing Significant Residual Mitral Regurgitation After Successful Deployment of the First XTW MitraClip

Video 3 Echocardiographic and Fluoroscopic Videos of Various Stages Leading to Embolization of the Second XTW MitraClip Into the Right Lower Pulmonary Vein

Video 4 Echocardiographic and Fluoroscopic Videos of Various Stages of Transcatheter Retrieval of Embolized XTW MitraClip From the Right Lower Pulmonary Vein
